# Effect of Perceived Stress on Health-Related Quality of Life among Primary Caregiving Spouses of Patients with Severe Dementia: The Mediating Role of Depression and Sleep Quality

**DOI:** 10.3390/ijerph19137962

**Published:** 2022-06-29

**Authors:** Jinheum Kim, Eunjeong Cha

**Affiliations:** 1 Department of Applied Statistics, University of Suwon, Hwaseong-si 18323, Korea; jinhkim@suwon.ac.kr; 2 Department of Nursing Science, University of Suwon, Hwaseong-si 18323, Korea

**Keywords:** dementia, depression, sleep quality, perceived stress, primary caregiver, spouse, health-related quality of life

## Abstract

Recently, there has been a rise in the number of spouses becoming primary caregivers to patients with dementia. This study identifies the mediating effects of depression and sleep quality on the relationship between perceived stress and health-related quality of life (HRQoL) among primary caregiving spouses of patients with severe dementia through a secondary data analysis of the 2018 Korea Community Health Survey by the Korea Disease Control and Prevention Agency. Data from 229 primary caregiving spouses of patients with severe dementia were analyzed using descriptive statistics, Spearman’s rank correlation or Pearson’s correlation analysis, and the lavaan R package, version 0.6-9. The association between perceived stress status (PSS) and the European Quality of Life Five Dimension (EQ-5D) index was highly significant. The direct effect of PSS observed in the model was nullified with both the Patient Health Questionnaire-9 and the Pittsburgh Sleep Quality Index as mediators, which implies that they mediate the effect of PSS on caregivers’ EQ-5D indexes. The mediation model accounted for 33.2% of the variance in the EQ-5D index of caregivers. The results suggest the need to develop an intervention to improve sleep quality and manage depression to mitigate a decline in HRQoL for these caregivers.

## 1. Introduction

According to a report by the World Health Organization, approximately 50 million people worldwide currently live with dementia. Researchers predict that by 2050, 152 million people will develop dementia due to aging [1]. In Korea, the number of people diagnosed with dementia increased by approximately 30% in 2019 compared to 2010 and reached 959,000, with an increase observed every year [2]. Patients with dementia lose the ability to perform daily activities as the disease progresses and require intensive nursing and care [3]. Many patients with dementia and these dependent characteristics are cared for by their families [4,5]. The burden of caring for patients with dementia is multifaceted and extensive and includes daily difficulties with physical, mental, social, and financial family problems [6]. Recently, there has been an increase in the number of spouses becoming primary caregivers to patients with dementia due to changes in family values and structure, such as an increase in older-couple-only households [7]. When the spouse is the primary caregiver, they tend to feel a higher burden of caregiving than other family members [8]. This situation may cause more stress, as spouses’ age-related health problems and functional impairments interfere with providing care [9].

Nolan et al. [10] viewed the stress of a caregiver as the result of the perceived im-balance between the actual degree of demand on the person and the person’s capacity to accommodate. For caregivers, tension and stress are both the causes and effects of phenomena that break down an individual’s equilibrium, and from this point of view, subjective or perceived stress is more important than the actual factors of stress. The stress caused by caring for patients with dementia affects the physical and mental health and quality of life of family caregivers [11]. Health-related quality of life (HRQoL) is directly linked to an individual’s health. HRQoL is a subjective and multidimensional concept that helps evaluate the daily functioning and well-being of the elderly [12]. In previous studies, the HRQoL of a primary caregiver was found to be lower when the caregiver was the spouse [7]. However, many studies on the quality of life of families providing care for patients with dementia have not shown consistent results [10]. Therefore, it is necessary to examine the HRQoL factors that are connected with the perceived stress of the primary caregiver spouse of a patient with dementia.

Studies have revealed that depression is significantly correlated with perceived stress and quality of life [7]. In addition, perceived stress has been shown to influence depression [13]. In the case of the elderly, it has been reported that the stress of negative life events such as a spouse’s disease affects depression [14] and that this is due to a decline in health and a decrease in psychosocial resources due to aging [15]. In contrast, Valimaki et al. [16] found that depression was higher when the spouse was a caregiver and depression negatively affected HRQoL.

Researchers have also reported that quality of sleep is significantly correlated with perceived stress and HRQoL [17]. Stress has been shown to strongly affect sleep quality through physiological and behavioral response mechanisms [18], and sleep quality influences HRQoL [19]. Previous studies have found that the sleep quality of caregivers for patients with dementia is lower than that of noncaregivers [20,21]. The quality of sleep of caregivers is affected by various factors such as the behavior of patients with dementia and the age of caregivers, the elderly being more vulnerable to sleep deficiency [22]. In addition, it has been found that the overall health of the caregivers of patients with dementia is affected by sleep quality [23].

**Figure 1:**
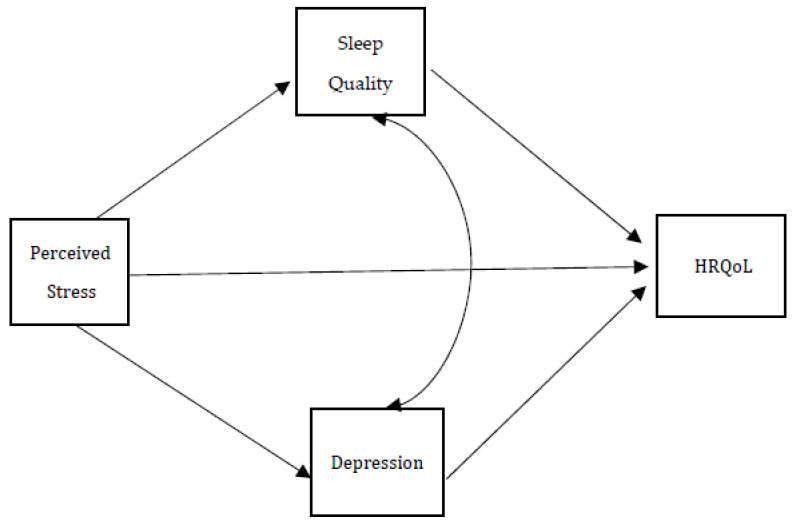
Hypothetical mediation models.

Research suggests that there is a strong bidirectional relationship between depression and quality of sleep [24,25]. These findings imply that depression and quality of sleep have mediating effects on the relationship between perceived stress and HRQoL. Therefore, the purpose of this study was to examine the mediating effects of depression and sleep quality on the effects of perceived stress on the HRQoL of the primary caregiver spouses of patients with severe dementia. Based on the above-mentioned studies, we hypothesize the following relationships between perceived stress and HRQoL (Figure 1):

**Hypothesis** **1:** *Perceived stress predicts HRQoL via depression.*

**Hypothesis** **2:** *Perceived stress predicts HRQoL via sleep quality.*

**Hypothesis** **3:** *Both depression and sleep quality mediate the relationship between perceived stress and HRQoL.*

## 2. Materials and Methods

### 2.1. Study Design and Data Source

We performed a secondary data analysis of the 2018 Korea Community Health Survey (KCHS) conducted by the Korea Disease Control and Prevention Agency (KDCA).

We visited the KCHS website (https://chs.kdca.go.kr, accessed on 29 October 2020) and requested to use the 2018 KCHS data for research. We disclosed information on the research, investigators, and the purpose of use and agreed to comply with the agency’s rules to download the 2018 KCHS raw data, questionnaire, and codebook.

### 2.2. Participants

We surveyed a total of 125,935 households. Among them, only 399 households consisted of couples in which the spouse was the primary caregiver of a patient diagnosed with dementia. The households of patients with mild dementia, which totaled 170, were excluded from analysis, as it was not possible to differentiate between the couple’s responses as both the caregiver and the dementia patient responded. Finally, the caregiver responses from 229 households of patients with severe dementia were analyzed.

### 2.3. Measurements

We defined the study variables based on the survey items of the 2018 KCHS by the KDCA as follows:

The sociodemographic characteristics included in this study were the genders, ages, educational levels, employment statuses, and residential areas of caregivers. Age was categorized into “<65,” “65–74,” “75–84,” and “≥85” years. The level of education was split into four categories: “uneducated,” “elementary school graduate,” “middle or high school graduate,” and “college or higher graduate.” The residential area was classified into “large” and “small” cities; “Dong” in Korean corresponds to the former and “Eup or Myeon” to the latter. Health behaviors included “drinking” and “walking.” For drinking, “no” was defined as lifelong abstinence from alcohol consumption or abstinence for the most recent year, while “yes” represented all other drinking patterns. For walking, “yes” was defined as practicing walking for ≥30 min and ≥5 days per week and “no” otherwise.

Perceived stress status (PSS) measures the amount of stress that a caregiver has experienced in their daily life during the past year (for this study: 2017–2018). The four-response Likert scale ranges from 0 (never) to 3 (very strong). PSS was recoded to “low” for 0 and 1 and “high” for 2 and 3 as a factor in the mediation models.

The Korean version of the Patient Health Questionnaire-9 (PHQ-9) measures the extent to which an individual experienced depression over the two weeks preceding the test [26]. The four-response Likert scale ranges from 0 (not at all) to 3 (nearly every day); the total scores range from 0 to 27, with higher scores indicating higher levels of depressive symptoms. In this study, the Cronbach’s α of all the items in the Korean version of the PHQ-9 was 0.84.

The Korean version of the Pittsburgh Sleep Quality Index (PSQI) measures the extent to which an individual experienced a wide variety of factors associated with quality of sleep and related disturbances over the past month [27]. Nineteen individual items create seven component scores: subjective sleep quality, sleep latency, sleep duration, habitual sleep efficiency, sleep disturbances, the use of sleeping medication, and daytime dysfunction. The sum of the scores for these seven components provides a total score. The Likert scale of the seven components ranges from 0 to 3, and the total score ranges from 0 to 21, with higher scores indicating a worse quality of sleep. The Korean version of the PSQI has been previously validated [27]. In this study, the Cronbach’s α of the seven component scores was 0.67.

The European Quality of Life Five Dimension (EQ-5D) measures the extent to which an individual is currently experiencing problems in mobility, self-care, usual activities, pain/discomfort, and anxiety/depression [28]. The three-response Likert scale options range from 1 (none) to 3 (severe problem). In this study, Cronbach’s α was 0.79, which showed that EQ-5D had excellent internal consistency. The formula for the EQ-5D index is: EQ-5D index  =  1 − (0.05  + 0.096 × m2  +  0.418 × m3  + 0.046 × sc2  +  0.136 × sc3  +  0.051 × ua2  +  0.208 × ua3  +  0.037 × pd2  +  0.151 × pd3  +  0.043 × ad2  + 0.158 × ad3  +  0.05 × n3), where m2 = 1 for mobility of level 2; otherwise, 0; m3 = 1 for mobility of level 3; otherwise, 0; sc2 = 1 for self-care of level 2; otherwise, 0; sc3 = 1 for self-care of level 3; otherwise, 0; ua2 = 1 for usual activities of level 2; otherwise, 0; ua3 = 1 for usual activities of level 3; otherwise, 0; pd2 = 1 for pain/discomfort of level 2; otherwise, 0; pd3 = 1 for pain/discomfort of level 3; otherwise, 0; ad2 = 1 for anxiety/depression of level 2; otherwise, 0; ad3 = 1 for anxiety/depression of level 3; otherwise, 0; n3 = 1 for at least one of level 3; otherwise, 0. The EQ-5D index ranged from −0.171 to 1, with higher indexes indicating higher quality of life. The Korean version of the EQ-5D has been validated by the KDCA [29].

### 2.4. Ethical Considerations

KCHS data are openly published and the participants’ data were anonymized prior to their release. This study was approved by the Institutional Review Board at the university to which researchers belong (USW IRB/2004-045-01).

### 2.5. Data Analysis

First, a frequency analysis of the sociodemographic characteristics, health behaviors, and PSSs of the study subjects was performed.

Second, the associations between variables were tested through Pearson’s correlation analysis or Spearman’s rank correlation analysis. To satisfy the assumptions of the mediation models, there needed to be a statistically significant correlation between the independent variable (PSS), the mediators (PHQ-9 score and PSQI), and the dependent variable (EQ-5D index) [30].

Third, four regression models were used to test the three hypotheses stated earlier. In Model 1, the effect of PSS on the EQ-5D index was measured after adjusting for sociodemographic characteristics and health behaviors. Model 2 assessed the degree to which the PHQ-9 score mediated the relationship between PSS and the EQ-5D index. Model 3 assessed the degree to which PSQI mediated the relationship between PSS and the EQ-5D index. Finally, Model 4 assessed the degree to which both the PHQ-9 score and PSQI score simultaneously mediated the relationship between PSS and the EQ-5D index. Models 2, 3, and 4 tested Hypotheses 1, 2, and 3, respectively. All four models were implemented using the R lavaan package, version 0.6-9 [31]. Model fit was assessed using the chi-squared test statistic, root-mean-square error of approximation (RMSEA) with a cut-off value of 0.1 [32], standardized root-mean-square residual (SRMR) with a cut-off value of 0.1 [33], goodness-of-fit index (GFI) with a cut-off value of 0.9, and adjusted goodness-of-fit index (AGFI) with a cut-off value of 0.9 [34]. In addition, the statistical significance of the direct and total effects of PSS on the EQ-5D index, as well as the indirect effects of PHQ-9 and PSQI, were tested through 1000 bootstrap samples. We also provided 95% bias-corrected percentile bootstrap confidence intervals for the size of each effect.

## 3. Results

### 3.1. Participants’ Sociodemographic Characteristics

Table 1 summarizes the sociodemographic characteristics, health behaviors, PSSs, PHQ-9 scores, PSQIs, and ED-5D indexes of the participants. Of the 229 participants, 62.5% were female; 10.4% were under 65 years old, 28.0% were 65–74 years old, 52.0% were 75–84 years old, and 9.6% were 85 years old or older; 66.4% were unemployed; and 65.5% were living in large towns. Regarding the education level of the participants, 21.8% were uneducated, 45.4% graduated from elementary school, 27.5% graduated from middle or high school, and 5.3% graduated from college or higher. Of the participants, 58.1% did not drink alcohol and 64.2% did not walk regularly. Among the participants, 52.8% responded that they had high PSS. The overall means (standard deviation) of the PHQ-9 scores, PSQIs, and EQ-5D indexes were 4.68 (5.25), 7.35 (3.89), and 0.81 (0.18), respectively.

### 3.2. Correlation between Sociodemographic Characteristics, Health Behaviors, PSS, PHQ-9 Score, PSQI, and EQ-5D Index

As shown in Table 2, sociodemographic characteristics except for town and health behaviors were not significantly associated with the PHQ-9 score, and sociodemographic characteristics except for employment and health behaviors were not significantly associated with PSQI. However, sociodemographic characteristics, except for gender and town, and health behaviors were significantly related to the EQ-5D index. There were significant associations between PSS, PHQ-9 score, PSQI, and the EQ-5D index, indicating that mediation models among these four variables could be considered.

### 3.3. The Mediating Effects of the PHQ-9 Score and PSQI on the Relationship between PSS and the EQ-5D Index

Table 3 summarizes the results of the mediation models that analyzed the effect of PSS on the EQ-5D index after adjusting for sociodemographic characteristics and health behaviors. We used the bootstrap method to estimate the standard errors of the total, direct, and indirect effects. While the *p*-value is calculated by approximating the standard normal distribution, the lower and upper limits of the 95% bootstrap confidence interval are calculated by simultaneously correcting the bias and standard error. Therefore, when the *p*-value has a value around 0.05, the two results can be inconsistent with each other.

First, Model 1 tested whether the effect of PSS on the EQ-5D index was statistically significant after adjusting for sociodemographic characteristics and health behaviors. The goodness-of-fit statistics indicated that Model 1 fit the data well: χ^2^ = 9.61, df = 11, *p* = 0.566; RMSEA < 0.001, SRMR = 0.024, GFI = 0.963, and AGFI = 0.690. The total effect (direct effect) of PSS on the EQ-5D index of caregivers was −0.076, which was highly statistically significant (*p* < 0.001). Model 1 explained 28.3% of the variation in the EQ-5D index of caregivers.

Second, Model 2 added the PHQ-9 score as a mediator to Model 1 to test Hypothesis 1. The goodness-of-fit statistics indicated that Model 2 fit the data well: χ^2^ = 36.54, df = 22, *p* = 0.027; RMSEA = 0.054, SRMR = 0.038, GFI = 0.911, and AGFI = 0.573. In fact, since the anxiety/depression dimension of EQ-5D is positively correlated with the PHQ-9 score (Pearson’s correlation coefficient r = 0.52, *p* < 0.001), it was expected that the PHQ-9 score would function as a mediator in the relationship between PSS and HRQoL. The direct effect of PSS on the EQ-5D index of caregivers was reduced to -0.045 compared to that of Model 1, which corresponds to a reduction of 40.8%. However, the effect remained close to significance (*p* = 0.061). Model 2 explained 31.9% of the variation in the EQ-5D index of caregivers.

Third, Model 3 added the PSQI as a mediator to Model 1 to test Hypothesis 2. The goodness-of-fit statistics indicated that Model 3 fit the data well: χ^2^ = 23.97, df = 22, *p* = 0.349; RMSEA = 0.020, SRMR = 0.035, GFI = 0.938, and AGFI = 0.704. The direct effect of PSS on the EQ-5D index of caregivers was reduced to −0.053 compared to that of Model 1, which corresponds to a reduction of 30.3%. However, the effect remained highly significant (*p* = 0.015). Model 3 explained 31.1% of the variation in the EQ-5D index of caregivers.

Fourth, Model 4 simultaneously added the PHQ-9 score and PSQI as mediators to Model 1 to test Hypothesis 3. The goodness-of-fit statistics indicated that Model 4 fit the data well: χ^2^ = 46.80, df = 33, *p* = 0.056; RMSEA = 0.043, SRMR = 0.044, GFI = 0.912, and AGFI = 0.682. Further, the estimated covariance between the PHQ-9 score and the PSQI was 6.54 (bootstrap-based 95% confidence interval = [4.40, 9.33]), and the association test statistic was 5.34 (*p* < 0.001), indicating that the dependency between them as shown in Figure 1 is statistically valid. The magnitude of the indirect effect of PSQI on the EQ-5D index was similar to that of the PHQ-9 score (−0.023 vs. −0.017), and their *p*-values were also similar (0.056 vs. 0.050). Due to the indirect effects of these two mediators, the direct effect of PSS on the EQ-5D index was no longer statistically significant (*p* = 0.108). This suggests that the PHQ-9 score and PSQI mediated the effects of PSS on the EQ-5D index of caregivers. Model 4 explained 33.2% of the variation in the EQ-5D index of caregivers.

## 4. Discussion

The purpose of this study was to provide fundamental data for developing an intervention program to improve the HRQoL of primary caregiving spouses of patients with dementia by confirming the mediating effects of depression and sleep quality on the relationship between perceived stress and HRQoL.

Examining the general characteristics of the primary caregiver spouses of patients with dementia in this study, we found that 89.6% of the participants were older than 65 years. Of the participants, 52.8% responded that their perceived stress was high, as compared with the reported 25.4% [17] and 27.2% [35] for all age groups. The value in the current study was higher than that established by Jang and Han [7], who reported a value of 42.7% among spouses who were the primary caregivers of patients with dementia. The participants’ average score for depression (PHQ-9) was 4.68. This result was higher than the scores of the elderly living alone in the community (4.25) and the scores of the not-living-alone elderly in the community (2.86) [13]. The average score of sleep quality (PSQI) was 7.35, therefore indicating that the participants generally experienced poor sleep quality. These results are higher than the score of 5.63 reported in a study with community-dwelling adults [17]. In addition, it showed similar results to the previously reported 7.45 for caregivers of patients with dementia [23]. These results are supported by research results that in the case of the elderly caring for patients with dementia, various factors, such as the age of the caregiver and the night’s sleep variability due to caring for the patient with dementia, may be reflected [21]. The average HRQoL (EQ-5D) of the participants in this study was 0.81. The average HRQoL of the participants of a previous study [7] targeting spouses with dementia was 0.74, and that among the general population was 0.91 [36]. Based on these results, we can confirm that the spouses who are primary caregivers of patients with dementia have relatively high levels of perceived stress and depression, poor quality of sleep, and low HRQoL.

In this study, the participants’ perceived stress had a direct effect on HRQoL, which supports the findings of previous research [37,38]. When depression was introduced as a mediator between perceived stress and HRQoL, the direct effect of perceived stress on HRQoL decreased. However, the amount of variance in the participant’s quality of life explained by the model increased. In a previous study [39], depression had a mediating effect on the relationship between perceived stress and quality of life of college students. It was also established that depression partially mediates the relationship between job stress and quality of life for firefighters [40]. The prevalence of depression—a life-threatening factor—among older persons in Korea is more than four times that of adults under the age of 64 [41]. In the case of the primary caregiver spouses of patients with dementia, it is expected that the deterioration of their HRQoL may be prevented by the early detection of and intervention against depressive symptoms. These caregivers were found to be vulnerable to depression due to the stress induced by caregiving. When sleep quality was introduced as a mediator, the direct effect of perceived stress on HRQoL decreased. Nevertheless, it remained statistically significant, the explanatory power of the participants’ quality of life increased. This fact can be attributed to the mediating effect of sleep quality on the relationship between environmental factors and quality of life for adults aged 20 and over [42]. These results suggest that perceived stress enhances the explanatory power of HRQoL through sleep quality. Finally, when depression and sleep quality were added as mediators to the parallel model, both variables showed full mediating effects on perceived stress and HRQoL.

A key difference of this study is that while subjective stress, depression, and sleep quality were previously identified simply as factors influencing the HRQoL of participants, here the mediating effects of depression and sleep quality between subjective stress and HRQoL has been verified. As for these results, it is important to detect caregivers’ depression and poor sleep quality early in order to improve their quality of life. In addition, it is necessary to find an intervention plan to reduce depression and improve sleep quality. Drug therapy and nondrug therapy are mainly applied as sleep-related interventions. Drug therapy has the advantage of being effective in a short time but has the disadvantage of causing many side effects, such as a decrease in deep sleep time, interaction with combination drugs, physical and psychological dependence on drugs, and increase in falls and mortality [43]. Therefore, the first line of treatment should prioritize a combination of sleep hygiene intervention and cognitive-behavioral therapy. Recently, other combination therapies have been proposed, for example, bright light therapy with melatonin, auditory stimulation, and noninvasive brain stimulation (NiBS), as promising interventions [44].

This study has the following limitations to be considered for future research. First, the number of participants was not sufficient to classify perceived stress into more than two categories. Hence, the analysis was undertaken by dividing perceived stress into “high” and “low” categories. Second, due to the limited nature of the secondary data used in our study, variables related to caregiver burden, the characteristics of patients with dementia, and variables on social support for dementia patient care could not be included in the analysis. Third, research based on self-reporting data may be somewhat unreliable, as nearly 90% of the participants are over the age of 65, a time when cognitive function begins to decline. Fourth, although the KCHS study has been refined by conducting surveys annually over 15 years from 2008, the results of this study may be limited because they are survey data for multipurpose research.

## 5. Conclusions

This study found that the primary caregiving spouses of patients with severe dementia were vulnerable to subjective stress, depression, low sleep quality, and low HRQoL. In addition, based on the fundamental data provided by this study, it is necessary to facilitate social interest and practical measures to manage depression and improve the sleep quality of the primary caregivers of patients with dementia. This step will mitigate the deterioration of the HRQoL of the primary caregiver spouses of patients with severe dementia due to perceived stress.

## Figures and Tables

**Table 1:** Descriptive statistics of participants’ sociodemographic characteristics, health behaviors, PSSs ^1^, PHQ-9 ^1^ scores, PSQIs ^1^, and EQ-5D ^1^ indexes.

Variables	Categories	*n* (%) or M ± SD ^1^
Total	-	229 (100)
Gender	Male	86 (37.5)
	Female	143 (62.5)
Age	<65	24 (10.4)
	65–74	64 (28.0)
	75–84	119 (52.0)
	≥85	22 (9.6)
Education	Uneducated	50 (21.8)
	Elementary	104 (45.4)
	Middle or high	63(27.5)
	College or higher	12(5.3)
Employment	No	152 (66.4)
	Yes	77 (33.6)
Town	Small to medium	79 (34.5)
	Large	150 (65.5)
Drink	No	133 (58.1)
	Yes	96 (41.9)
Walk	No	147 (64.2)
	Yes	82(35.8)
PSS	Low	108 (47.2)
	High	121 (52.8)
PHQ-9 score	-	4.68 ± 4.80
PSQI	-	7.35 ± 3.89
EQ-5D index	-	0.81 ± 0.18

^1^ PSS = perceived stress status; PHQ-9 = Patient Health Questionnaire-9; PSQI = Pittsburgh Sleep Quality Index; EQ-5D = European Quality of Life Five Dimension; M = mean; SD = standard deviation.

**Table 2:** Correlation analysis ^1^ between sociodemographic characteristics, health behaviors, PSS ^2^, PHQ-9 score ^2^, PSQI ^2^, and EQ-5D index ^2^.

Variables	Gender	Age	Education	Employment	Town	Drink	Walk	PSS	PHQ-9 Score	PSQI	EQ-5D Index
Gender	-	<0.001	0.001	0.756	0.704	0.002	0.611	0.349	0.995	0.477	0.678
Age	−0.33	-	0.011	<0.001	0.866	0.856	<0.001	0.212	0.679	0.255	0.002
Education	−0.26	−0.17	-	0.939	<0.000	0.092	0.219	0.765	0.167	0.185	0.002
Employment	−0.02	−0.29	−0.01	-	<0.001	0.182	0.198	0.454	0.195	0.023	<0.001
Town	−0.03	0.01	0.32	−0.22	-	0.553	0.025	0.367	0.007	0.185	0.387
Drink	−0.20	−0.01	0.11	0.09	−0.04	-	0.065	0.467	0.477	0.188	0.013
Walk	0.03	−0.24	0.08	0.09	0.15	0.12	-	0.646	0.675	0.343	<0.001
PSS	0.06	−0.08	0.02	−0.05	0.06	−0.05	0.03	-	<0.001	<0.001	0.014
PHQ-9 score	0.00	−0.03	−0.09	−0.09	0.18	0.05	−0.03	0.33	-	<0.001	<0.001
PSQI	0.05	0.08	−0.09	−0.15	0.09	−0.09	−0.06	0.24	0.50	-	<0.001
EQ-5D index	0.03	−0.21	0.20	0.27	−0.06	0.16	0.25	−0.16	−0.37	−0.38	-

^1^ The entries under the diagonal represent Pearson (or Spearman) correlation coefficients and those above the diagonal represent *p*-values for the respective correlation. ^2^ PSS = perceived stress status; PHQ-9 = Patient Health Questionnaire-9; PSQI = Pittsburgh Sleep Quality Index; EQ-5D = European Quality of Life Five Dimension.

**Table 3:** Mediation analysis ^1^ of PSS ^2^ on the EQ-5D index ^2^ through the PHQ-9 score ^2^ and PSQI ^2^ as mediators.

						95% CI ^2,3^		Proportion	
Model	Mediator (s)	Effect	Estimate	Z ^3^	*p*-Value ^3^	LL ^1^	UL ^1^	(%)	R ^2^
1	-	Total	−0.076	−3.51	<0.001	−0.119	−0.034		0.283
2	PHQ-9 score	Total	−0.076	−3.57	<0.001	−0.118	−0.036		0.319
		Direct	−0.045	−1.87	0.061	−0.095	−0.003		
		Indirect	−0.032	−2.81	0.005	−0.058	−0.012	42.1	
3	PSQI	Total	−0.076	−3.56	<0.001	−0.120	−0.035		0.311
		Direct	−0.053	−2.43	0.015	−0.098	−0.014		
		Indirect	−0.023	−2.65	0.008	−0.044	−0.008	30.3	
4	PHQ-9 score,	Total	−0.076	−3.60	<0.001	−0.115	−0.035		0.332
	PSQI	Direct	−0.036	−1.61	0.108	−0.082	0.008		
		Indirect (All)	−0.040	−3.36	0.001	−0.068	−0.021	52.6	
		Indirect (PHQ-9)	−0.023	−1.91	0.056	−0.052	−0.003	30.3	
		Indirect (PSQI)	−0.017	−1.96	0.050	−0.040	−0.004	22.4	
		Model							
		1		2		3		4	
Variates	Categories	β	*p*-value	β	*p*-value	β	*p*-value	β	*p*-value
Gender	Female	0.054	0.025	0.044	0.085	0.055	0.041	0.048	0.060
Age (ref: <65)	65–74	0.001	0.987	−0.006	0.798	0.008	0.750	0.001	0.977
	75–84	−0.044	0.238	−0.058	0.026	−0.036	0.140	−0.048	0.055
	≥85	−0.003	0.955	−0.016	0.677	0.013	0.753	−0.001	0.975
Education	Elementary	−0.037	0.183	−0.04	0.113	−0.035	0.150	−0.038	0.140
(ref:	Middle or high	0.049	0.140	0.034	0.261	0.042	0.139	0.033	0.279
Uneducated)	College or higher	0.179	0.001	0.147	<0.001	0.154	0.001	0.137	0.002
Employment	Yes	0.069	0.003	0.060	0.003	0.059	0.005	0.056	0.007
Town	Large	−0.045	0.057	−0.018	0.518	−0.034	0.140	−0.018	0.514
Drink	Yes	0.032	0.139	0.041	0.030	0.029	0.150	0.036	0.060
Walk	Yes	0.085	<0.001	0.072	0.001	0.078	<0.001	0.070	<0.001

^1^ Results with adjustment for the effects of covariates: socio-demographic characteristics (gender, age, education, employment, and town) and health behaviors (alcohol consumption and walking). ^2^ PSS = perceived stress status; PHQ-9 = Patient Health Questionnaire-9; PSQI = Pittsburgh Sleep Quality Index; EQ-5D = European Quality of Life Five Dimension; CI = confidence interval; LI = lower limit; UI = upper limit. ^3^ Bootstrap-based values with 1000 resamples.

## Data Availability

The data that support the findings of this study are available from the corresponding author upon reasonable request.
